# Scarlatina or Something Similar*Read at the Palestine Meeting of the East Texas Medico-Chirurgical Society, July 6, 1900.

**Published:** 1900-09

**Authors:** W. T. Evans

**Affiliations:** Jewett, Texas


For the Texas Medical Journal.

### Scarlatina or Something Similar.*

W. T. EVANS, M. D., JEWETT, TEXAS.

When I received notice that I was on the program and would be expected to read a paper on some medical subject at this meeting, I began to revolve in mind the various subjects with which I was most familiar, in order to select one that would be interesting and instructive and about which I could say the most; but realizing the fact, that this body is composed of men of learning and experience, and feeling that I was always more willing to receive than to give instructions, I concluded to select a subject of which I know but little and that little, only from theory. For, during my many years of practice and varied experience with many forms of disease, if I have ever seen a case of scarlatina outside of the hospital, I do not know it. The case I report in this paper may have been one; if it was not, I must confess I do not know what it was. I have not yet made out diagnosis. When I am called to see a patient, my first object is to try to arrive at a correct diagnosis. In this case, however, my mind has not been fully satisfied, though the little sufferer has long since gone to that bourne from which no traveler ever returns. I say I do not know whether I was treating a

---

*Read at the Palestine Meeting of the East Texas Medico-Chirurgical Society, July 6, 1900.

case of scarlet fever, or not. It it rather late to find out now; too late to be any benefit to the patient, but like a post-mortem examination, it may benefit the living to discuss the case,—if not the dead; hence I bring it before you and ask a consultation.

I had no trouble in arriving at a correct prognosis, and I informed the parent of my opinion and suggested that they call in my friend, Dr. S. R. Burroughs, but they seemed satisfied that I should manage the case alone.

I know, that any intelligent physician who can understand the English language, can read the various authors, can post himself and be able to diagnose many diseases, with which he had not before came in contact, and by extensive reading and compiling and copying from said authors, can produce a more interesting and instructive paper than this; but I believe in these "experience meetings,"—believe in our relating the practical occurrences in daily practice. Hence I have not tried to theorize and elaborate, to show how much could be said on this subject, for I am satisfied you all are familiar with the theory and with what has been written in the medical books, concerning the same. In fact the object of the writer is not to tell so much about scarlet fever as to find out more about the "something similar." Thinking, perhaps, some of you may have had some experience on this line, I hope to call you out, or elicit some discussion so I may learn something.

In reading the medical journals and hearing from other physicians, I find there has been prevailing, in different parts of the State, an eruptive fever, not fully understood by all who treated it. I had several other cases myself—considered by others to be scarlet fever; but this one case herein reported, the only one in my practice that caused me any doubt as to diagnosis. Is it, or is it not, scarlet fever. Still I am not able to decide.

I now report the case: I was called to see a girl 7 or 8 years old, on Wednesday, December 13, 1899. I obtained the following history: On Saturday night previous (which was the 9th), she retired, complaining with headache, feeling chilly and had some fever, which continued; and on Monday the family discovered a rash or eruption on her neck and body. Tuesday they sent for me, but being absent from home, I did not see her until Wednesday. I found her very sick indeed; high fever, temperature 104 and 105; pulse very weak and frequent, general depression or prostration; she was semi-delirious; had earache, accompanied with partial deafness; increased redness and irritation of throat, involving uvula, margin of soft palates and tonsils; some pharyngitis. The catarrhal trouble seemed to be confined to the upper air passages; not affecting the lungs, though she had slight cough. The buccal and faucial mucous membranes presented a general red appearance; there was some slight fibrinous exudation, especially over the tonsils. No suffusion or irritation of eyes, and no undue lachrymation;—in fact, eyes seemed rather bright and clear; a peculiar looking tongue, covered at first with moist coating and rather white; the papillæ very red and prominent, also the borders or edges of tongue red; Schneiderian membrane participating in the general irritation or inflammatory process, giving off an irritating discharge, the surface over which it passed, at or near the nasal openings, became covered with the exudation or fluid, leaving a smooth surface. Before I was through with my examination, in fact before I began it, I could see the eruption on the neck, just above her clothing, and was under the impression the same had been produced by some irritant; in fact, it looked like it had been burned with mustard. On examination, I found the entire body presented the same appearance, a continued and uniform rash, even down to the lower extremities, and they were covered with a papular eruption. The hyperæmia of the skin over the body was very great, readily disappeared upon pressure and returned very rapidly when the pressure was removed; placing or tracing the finger over the parts, the skin would appear white, but very soon resume the scarlet hue. The exudation about the mouth was so tenacious that strings or bands of mucus would extend from tongue and lower gums to roof of mouth, when opened, and it could only be removed with difficulty. There was a feeling of fullness and roughness over the body, but no tumefaction or swelling of feet and hands. She died on the seventh day of her illness.

When I first saw the case,—before a thorough and careful examination, I did not know whether I had meningitis, roseola or scarlatina to treat. I soon became satisfied, however, that I did not have either of the first two,—but I do not know yet that I had a case of scarlet fever, and I wish some of you would tell me. At the time I did not have the benefit of the opinion of a consultant, but I was not entirely without some one to give an opinion and make suggestions; for Captain Nettles, who commanded the Val Verde battery during the Confederate war, and who is well known by some members present, to be a man who reads a great deal, in fact, does not do much else, informed me that he knew all about scarlet fever, and had had much experience in nursing patients afflicted with same—had seen many cases in the Old States, and during the war and upon the high seas, while he was in the navy, etc., and he was satisfied that the case under consideration was one of genuine scarlet fever and seemed surprised that I hesitated to give a positive diagnosis. I reminded him that scarlet fever is a contagious disease and that this child had not been exposed to the infection; had not been away from home, and as far as I knew, or could ascertain, had no possible chance for infection, and that therefore I would withhold a possitive opinion as to diagnosis until some other member of the family contracted the disease. There were several younger children, and some of the neighbor ladies who came to nurse and sit up with the sick, brought their babies, but not one became infected, and there were no precautions used, only I advised the visitors not to expose their babies unnecessarily. I am withholding my diagnosis yet. Had there been other cases, my doubts would have been removed, for I am of the opinion that fifteen out of twenty cases would not develop more fully the symptoms characteristic of the disease.

If this was scarlet fever (and I must say it resembled that more than anything else with which I am familiar), it seems to me there would have been a point of infection, or admitting that it may have been sporadic (all things have a beginning); then it appears that there would have been some cases among those children, who were on and about the bed frequently.

I have not said anything about the treatment and I will not do so until I am convinced what the disease was.

